# Renal cell carcinoma in the pediatric population: A case report and review of the literature

**DOI:** 10.1016/j.eucr.2023.102453

**Published:** 2023-06-02

**Authors:** Antonios Tawk, Rawad Abou Zahr, Khalil Chalhoub, Samer Danaf, Mohammed Hussein Kamareddine, Joe Nohra

**Affiliations:** a Faculty of Medicine and Medical Sciences, University of Balamand, Aschrafieh, Beirut, Lebanon; b Department of Urology, Saint George Hospital University Medical Center, University of Balamand, Beirut, 1100 2807, Lebanon

## Abstract

Renal cell carcinoma is a cancer thought to originate from renal epithelial cells. Commonly seen in patients older than 60 years of age, renal cell carcinoma presents as rare pathological entity seen in urological cancers among the pediatric population. A 17-year-old female patient presented with complaints of intermittency, dysuria, and gross hematuria. Radiological imaging was in favor of a left renal mass. Under general anesthesia, the left kidney was completely laparoscopically resected and sent to pathology, which along with correlating the age group of the patient and the morphology on pathological analysis, was suggestive of microphthalmia family translocation renal cell carcinoma.

## 1 Introduction

Renal cell carcinoma (RCC) is a tubular epithelial malignancy, most commonly occurring in patients between the ages of 50–70 years. Renal tumors account for 4–5% of all malignancies in those aged under 20 years; and RCC comprises 1–12.5% of all renal malignancies in the pediatric population. Silberstein et al. demonstrated that the age-adjusted incidence of RCC is 0.01/100,000. In the pediatric population, RCC is seen at a mean age of 10 years with a male to female ratio of 1:1.^1^

Although RCC is associated with the classic triad of gross hematuria, flank mass, and flank pain, less than 10% of RCC pediatric patients present with this triad.^2^

Children do not share the same RCC risk factors as their adult counterparts. Additionally, there are histological and genetic differences between adult and pediatric RCC. Studies using immunohistochemical methods have demonstrated that 20–70% (vs 0.2–4.2% of adults) of pediatric RCC displayed translocations involving the Xp11.2 and 6p21 loci. The Xp11.2 translocation, which involves the transcription factor TFE3 gene, is the most common translocation seen in pediatric RCC.^3^

Herein, we report a case of RCC in a 17-year-old female patient.

### 1.1 Case presentation

A 17-year-old female with a negative past medical history presented for abdominal pain, dysuria, gross hematuria, and intermittency. The patient and her non-consanguineous parents denied any family history of renal diseases. The patient reported sudden onset epigastric and left flank pain 40 days prior to her current presentation. The pain was followed by intermittent symptoms of gross hematuria and dysuria. The patient denied any traumatic event that might explain the gross hematuria. Subsequently, she was admitted to a peripheral hospital where initial workup was suspicious for a left renal mass. There, the patient underwent cystoscopy and ureteroscopy under general anesthesia; and a double-J was inserted on the left. The patient was later admitted to our facility for further evaluation and management.

**Fig. 1:**
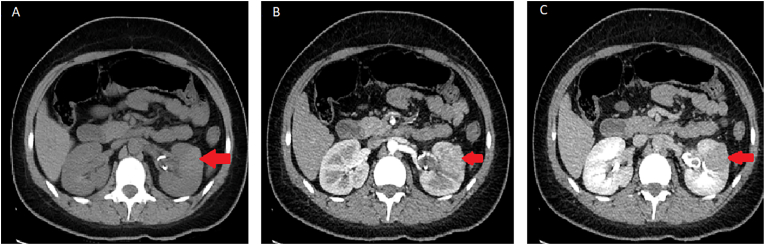
Enhanced CT scan of left kidney lesion in (A) non contrast phase, (B) arterial phase, and (C) nephrogenic phase. The red arrows point at kidney mass, which shows enhancement of the lesion with contrast. *The mass originates from the cortex measures 30 × 25 × 35 mm in the mid third of the left kidney. (For interpretation of the references to colour in this figure legend, the reader is referred to the Web version of this article.)

Abdomen CT scan with IV contrast showed a soft tissue attenuation mass measuring 3 × 2.5 × 3.5 cm in the anterior aspect of the middle third of the left kidney, originating from the cortex, extending to the underlying pyramids. There was no vascular extension to be noted but, a clot was seen in the renal pelvis and the inferior renal calyx. The right kidney was unremarkable. Few para-aortic and right paracolic sub-centimetric lymph nodes measuring up to 5 mm were noted (Fig. 1). This primary lesion of the left kidney was noted to be highly suspicious, and lymphoma could not be rule out. Hence the decision for a biopsy was taken.

The biopsy was performed under ultrasound guidance with pathology describing appearances compatible with RCC. MIT family translocation RCC was suggestive giving the age group and morphology. However, this hypothesis could not be confirmed due to the unavailability of TFE3 antibody in Lebanon.

Laparoscopic radical left nephrectomy was performed under general anesthesia. The left kidney, left ureter, Gerota's fascia, and the paraaortic lymph nodes were sent for pathological study. The kidney, measuring 11.5 × 7x4.5cm showed an attached fragment of fat measuring 8 × 3.5 × 1cm previously trimmed, showing a large hemorrhagic focus measuring 4 × 4 × 0.5cm. There is also a previously ruptured focus of the capsule overlying the tumor, measuring 2.5 × 1cm on the anterior aspect of the lower pole. There was no evidence of invasion of the perirenal fat. The renal sinus fat was intact. Background renal parenchyma was unremarkable. Sections of the renal tumor showed a neoplastic proliferation of papillary structures and acini lined by voluminous, columnar to polygonal cells with a clear or eosinophilic cytoplasm. The nuclei were enlarged, round to polygonal hyperchromatic with a prominent nucleolus. Psammomatous calcifications were present focally. Moreover, there was no evidence of neoplastic lymphovascular invasion, invasion into the perirenal fat or the sinus fat tumor necrosis but extensive interstitial hemorrhage. However, one metastatic lymph node was identified. The renal vein was unremarkable, and the distal ureteral margin was negative for tumor.

Postoperative histological studies confirmed RCC (stage pT1a) and it is most probably corresponding to translocation-associated subtype. The patient's postoperative course was uneventful, and she was discharged on the third postoperative day.

## 2 Discussion

RCC has four major growth patterns sarcomatoid, solid, papillary, and cystic, with the papillary pattern being predominant in the pediatric population as seen in our patient. The papillary pattern can arise from pre-existing malignancies, such as Wilms tumor.

CT scans help in differentiating between RCC from other malignancies but fail to provide a definitive diagnosis warranting histopathological and genetic studies.

Being inherently resistant to chemotherapy and radiotherapy, radical nephrectomy accounts for being the most common approach. The incomplete resection of the tumor results in survival rates as low as 10%.^4^ When compared to open radical nephrectomy, minimally invasive surgery is associated with shorter hospital stay and decreased need for narcotics and nasogastric tube. Many surgeons prefer laparoscopic for stage 1 or 2 RCC in which nephron-sparing surgery cannot be performed. While both approaches are equally effective, the laparoscopic approach is associated with lower morbidity.^5^ In the presented case, the patient underwent laparoscopic radical nephrectomy since more favorable outcome can be achieved while also taking into consideration the large hemorrhagic focus and previously ruptured focus of the capsule overlying the tumor.

Contrary to adult RCC, even small lesions in pediatric RCC may have positive lymph nodes. Hence, regional lymphadenectomy is advised with all primary RCC in young patients regardless of staging.^5^

Histopathology revealed 1 positive lymph node in our patient. She was placed on adjuvant therapy with Sunitinib, a multi-targeted receptor tyrosine kinase inhibitor.

For pediatric RCC staging, both the TNM system and the modified Robson staging classification system can be used. The overall 5-year survival rate is reported to be around 62%. Tumor stage appears to be the strongest prognostic indicator of survival in both population.^4^ In the presented case, postoperative histological studies revealed a stage pT1a RCC in which the patient's stage-specific 5-year survival would be above 92%.

The postoperative course was uneventful. At 6-month follow-up, the patient was unremarkable and was scheduled for a strict long-term follow-up.

## Consent

Informed consent from the patient and her parents was obtained for publishing this article and images.

## Declaration of competing interest

The authors declare no conflict of interest regarding the publication of this article.
